# Mixotrophic plankton foraging behaviour linked to carbon export

**DOI:** 10.1038/s41467-022-28868-7

**Published:** 2022-03-14

**Authors:** Natalie R. Cohen

**Affiliations:** Skidaway Institute of Oceanography, University of Georgia, Savannah, USA

**Keywords:** Carbon cycle, Carbon cycle

## Abstract

Marine mixotrophic protists that use both heterotrophic and phototrophic metabolisms may impact the carbon cycle in unexpected ways. A recently characterized mixotroph can craft three-dimensional mucilage feeding structures that trap nutrient-rich plankton prey and contribute to the sinking of carbon from the surface ocean.

Single-celled eukaryotes (protists) are microbial powerhouses that help drive the marine biological carbon pump, or the biologically driven vertical movement of carbon from surface waters to the ocean’s interior. Protists have diverse lifestyles and can play key roles in ocean carbon cycling through both autotrophic photosynthetic carbon fixation and heterotrophic respiration of carbon. Unlike other microorganisms that are solely autotrophs or heterotrophs, some protists are capable of mixotrophy, a combination of phototrophic and heterotrophic metabolisms^1,2^. Mixotrophs alter traditional views of how carbon flows through an ecosystem^3^. They enable more efficient trophic transfer of carbon up the food chain by offsetting respired carbon losses with photosynthesis^4^*.* Modeling suggests that this allows organisms in the ecosystem to be larger, and increases sinking of organic material through the biological carbon pump compared to a food web dominated by strict phototrophy and heterotrophy^4,5^. A recent global ocean gene sequencing survey suggests that mixotrophic lineages are ubiquitous, and they represent an estimated 12% of the microeukaryotic sequences recovered^6^. They are therefore hypothesized to be important contributors to carbon cycling across vast regions of the ocean. However, relatively few mixotrophs have been cultured in the lab^2^, and their specific ecological and biogeochemical roles largely remain a mystery.

Given their likely impact on biogeochemistry, inclusions of mixotrophs in global ocean carbon models may be important for improving carbon cycling predictions^7^. Carbon flux estimates from global circulation models do not consistently match experimentally derived rates^8^, and predictions could be strengthened by explicitly accounting for mixotrophic protists and their behaviour in biogeochemical and ecosystem models. In *Nature Communications*, Larsson et al.^9^ elegantly characterize a new mucilage feeding strategy in the mixotrophic dinoflagellate *Prorocentrum* cf*. balticum* (Fig. 1) and estimate the potential impact of this behaviour on the biological carbon pump, highlighting an additional mechanism by which mixotrophs may impact carbon biogeochemistry.

**Fig. 1:**
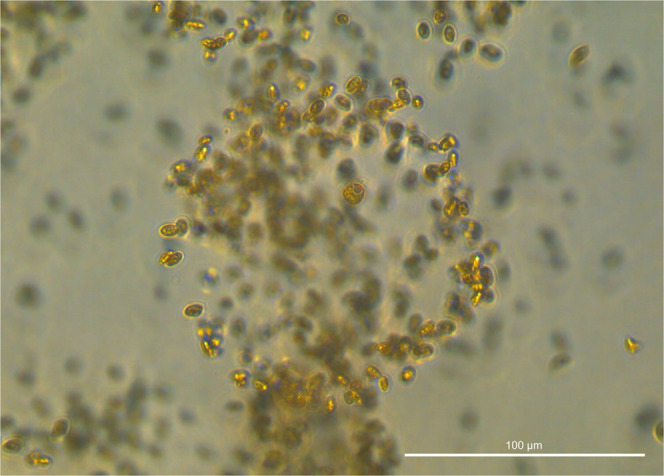
Mixotrophic feeding impacts the carbon cycle. The mixotrophic protist *Prorocentrum cf. balticum* (center) observed within the circular perimeter of its mucosphere, which is laden with captured Cryptophyte (*Rhodomonas salina*) prey cells. Abandoned mucospheres sink, contributing to ocean carbon sequestration. Dr. Michaela E. Larsson.

Mixotrophy is a flexible form of metabolism that allows protists to persist under various growth limiting conditions that can occur in marine environments. With mixotrophy, protists can utilize external nutrients to carry out photosynthesis and carbon fixation, or consume prey for carbon and/or nutrients when conditions are not conducive to photosynthesis^10^. The degree of phototrophic and heterotrophic metabolism exhibited in mixotrophs varies by organism, and shifts between trophic modes can be triggered by light, nutrients, prey abundance and quality, or other environmental factors^2,3^. In order to understand the drivers of these shifts in trophic modes, it is often necessary to culture mixotrophs in the lab, or observe them in their natural environment by performing experiments at sea.

Mixotrophy has been notoriously difficult to identify and quantify in the environment. Taxonomic affiliation cannot be reliably used to predict mixotrophy because species within a genus are not consistently mixotrophic^11^, and each species therefore needs to be individually assessed. Further complicating matters, detecting the presence of individual species known to be mixotrophic in a given environment does not mean both trophic modes are actively being used^2^, as physiology depends on a complex suite of environmental factors. In the field, mixotrophy has traditionally been evaluated by systematically confirming both the presence of photosynthetic plastids within cells as well as the ability to consume prey through fluorescent or radiolabeled assays^2^. If mixotrophs are able to be cultured in the lab, their physiology, feeding behaviour, and life cycles can be more easily interrogated, and their contributions to carbon and nutrient cycling in the ocean may become more clear.

Larsson et al. perform an exhaustive physiological analysis of the cultured protist *P*. cf. *balticum* and document its ability to capture prey using an extracellular, net-like mucous feeding structure that they call the mucosphere. Fluorescence and light microscopy images and video footage provide evidence for the mucosphere being used to trap prey (some many times larger than *P*. cf. *balticum* itself). This protist demonstrates incredible dexterity with its mucosphere, capable of physically moving it around, and is able to detach and leave behind the gelatinous trap after eating is completed. They use photosynthetically-derived energy to produce one mucosphere per day on average, and in turn use the nutrients obtained from prey to support their phototrophic growth. Culture experiments demonstrate that eukaryotic and prokaryotic prey are chemically attracted to this mucosphere, and the structures are mainly built when prey are present, with these interactions mediated by chemical cues.

The biogeochemical importance of this mixotrophic lifestyle occurs when feeding is finished. The authors observed that *P*. cf. *balticum* abandoned mucospheres after consuming only one prey cell. Because mucospheres are negatively buoyant, they sink once jettisoned by cells and could potentially contribute to particulate carbon sinking out of the surface ocean. Carbon flux associated with mucospheres was estimated using mucosphere production rates in culture, the carbon content contained within mucospheres, and minimum and maximum cell densities derived from time series sites. Their calculations suggest sinking carbon rates that range from 0.04–0.29 mg C m^−2^ d^−1^—increasing by approximately 4-fold if attached eukaryotes and prokaryotes are included in the estimation—and by 24-fold if the high cell densities under bloom conditions are considered. These maximum sinking carbon rates may rival those produced by much larger zooplankton which package fecal pellets and also discard gelatinous feeding structures and can be important vectors of organic carbon to the deep ocean^12^. Mucouspheres produced by *P*. cf. *balticum* and other protistan species with similar foraging behaviour could therefore be significant and previously overlooked contributors to carbon export.

This laboratory work represents an advancement in our understanding of mixotrophy and its role in the biological carbon pump. Importantly, it reminds us that microscopic observations of fascinating creatures and their extraordinary behaviours can yield globally-relevant insights. Findings by Larsson et al. raise several exciting questions and directions to be pursued in future studies. How do feeding and nutrient strategies differ in offshore, open ocean strains adapted to low nutrient conditions compared to the coastal counterpart used in this lab study? How widespread is this type of feeding strategy—do closely related taxa also use it? This is important to address, as the carbon flux estimates are based on cell densities derived from microscopic identifications that include species beyond *P*. cf. *balticum*. More broadly, this work has demonstrated the value in connecting cell behaviour to ocean scale cycling of energy, and future research will benefit from similar conceptual integrations between microbial ecology and biogeochemistry. To fine tune biogeochemical predictions, future efforts should prioritize collecting additional empirical data on mixotrophy at sea, quantifying population contributions to carbon cycling from geographically diverse regions, and continuing to probe the physiology of underexplored mixotrophs in culture.

Phytoplankton possess a range of abilities for acquiring nutrients in the diffuse ocean, with mixotrophy being a particularly advantageous and flexible strategy with multifaceted impacts on carbon biogeochemistry. In their publication, Larsson et al. characterize a mixotrophic feeding mechanism that may directly influence organic carbon export. Similar investigations into feeding behaviours and nutrient physiology of other underexplored species will be valuable for improving carbon cycling predictions, and other novel ecological strategies are almost certainly waiting to be discovered across protistan lineages.
